# Bacteriology of Epidemic of “Grippe” 1915-16

**Published:** 1916-10

**Authors:** 


**Bacteriology of Epidemic of "Grippe" 1915-16.** V. H. Moon and S. C. Summers, Indianapolis, Jour. Ind. State Med. Assn., Aug., in 17 cases examined by cultures from nasopharyngeal mucus, found no influenza bacilli but a green-producing, non-haemolytic streptococcus. No agglutinins were found in the blood of patients or convalescents but 5 showed a skin reaction indicating the persistence of anti-bodies. Mathers of Chicago, J. A. M. A., Jan. 1, found a haemolytic streptococcus in 17 of 24 cases; Rucker of Philadelphia, N. Y. Med. Jour., Feb. 12, found non-haemolytic streptococci in all of 20 cases, pneumococci and influenza bacilli being also demonstrated in two cases each.

